# Strategies and Considerations for Improving Recombinant Antibody Production and Quality in Chinese Hamster Ovary Cells

**DOI:** 10.3389/fbioe.2022.856049

**Published:** 2022-03-04

**Authors:** Jun-He Zhang, Lin-Lin Shan, Fan Liang, Chen-Yang Du, Jing-Jing Li

**Affiliations:** ^1^ Institutes of Health Central Plains, Xinxiang Medical University, Xinxiang, China; ^2^ Department of Biochemistry and Molecular Biology, Xinxiang Medical University, Xinxiang, China; ^3^ Henan International Joint Laboratory of Recombinant Pharmaceutical Protein Expression System, Xinxiang Medical University, Xinxiang, China

**Keywords:** recombinant antibody, Chinese hamster ovary cells, expression vector, glycosylation, genetic engineering

## Abstract

Recombinant antibodies are rapidly developing therapeutic agents; approximately 40 novel antibody molecules enter clinical trials each year, most of which are produced from Chinese hamster ovary (CHO) cells. However, one of the major bottlenecks restricting the development of antibody drugs is how to perform high-level expression and production of recombinant antibodies. The high-efficiency expression and quality of recombinant antibodies in CHO cells is determined by multiple factors. This review provides a comprehensive overview of several state-of-the-art approaches, such as optimization of gene sequence of antibody, construction and optimization of high-efficiency expression vector, using antibody expression system, transformation of host cell lines, and glycosylation modification. Finally, the authors discuss the potential of large-scale production of recombinant antibodies and development of culture processes for biopharmaceutical manufacturing in the future.

## Introduction

**FIGURE 1:**
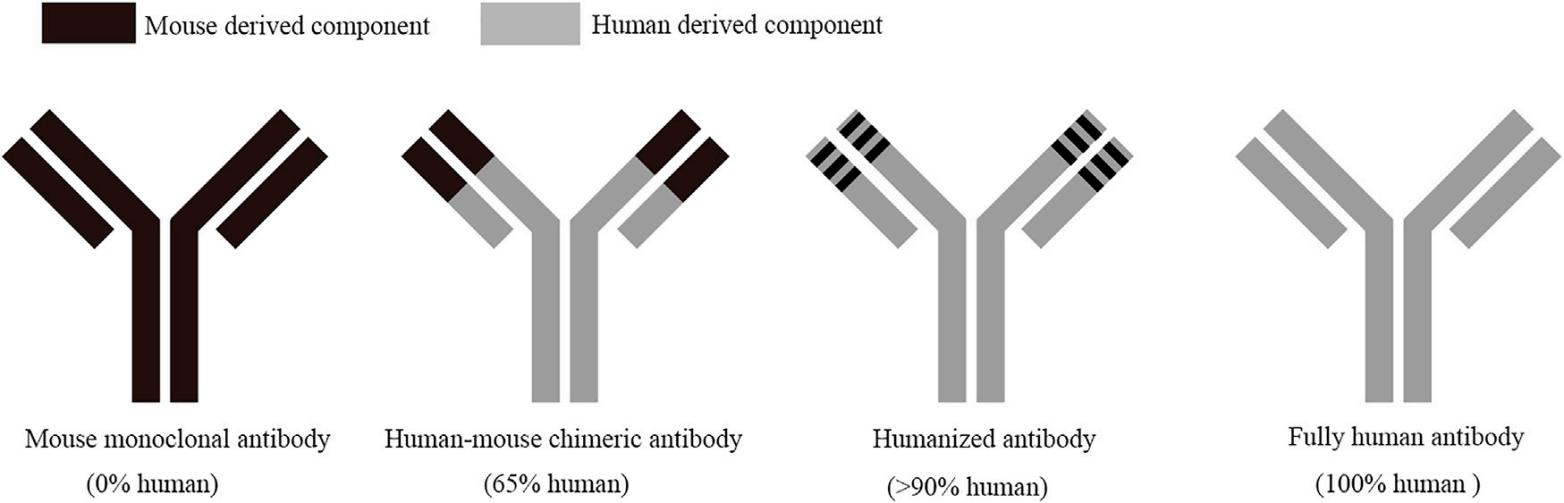
Development process of recombinant antibody drugs. Mouse monoclonal antibodies are all mouse-derived components, with large side effects and high immunogenicity; human-mouse chimeric antibodies are 65% human-derived components, with lower side effects than mouse monoclonal antibody; human-derived components in humanized antibodies are over 90%, with mild side effects and very low immunogenicity; fully human antibodies are all human-derived components, which can obviously remove the immunogenicity and side effects, and with good therapeutic effect.

In recent years, recombinant antibody drugs are emerging with the rapid development of modern molecular biology technology as well as in-depth exploration on the three-dimensional structure and mechanism of action of antibody molecules. Recombinant antibody drugs undergo the stages of mouse monoclonal antibody, human-mouse chimeric antibody, humanized antibody, and fully human antibody (Figure 1), which have been applied in many fields, such as anti-tumor, anti-autoimmune diseases, and biosensor (DeLuca et al., 2021; Galvani et al., 2021; Liao et al., 2021; Rudenko et al., 2021; Rybchenko et al., 2021; Yang G et al., 2021). Improving the affinity of antibodies and reducing their immunogenicity are two basic principles of genetic engineering of antibody drugs. The development of antibody drugs is relatively rapid. According to industry statistics, the global annual sales of antibody drugs in 1997 were only $300 million, more than $60 billion in 2012, exceeded $100 billion for the first time in 2017, and reached $123.2 billion in 2018. At present, antibody drug development has become one of the fastest growing, most profitable, and most feasible biopharmaceutical fields in the pharmaceutical industry; in particular, monoclonal antibodies have become one of the most important therapeutic recombinant antibodies in the global pharmaceutical market (Masuda et al., 2021; Savizi et al., 2021). Up to now, more than 300 biological drugs have been approved by the U.S. Food and Drug Administration (FDA), among which monoclonal antibodies are developing rapidly (Tihanyi and Nyitray, 2020). The production of recombinant antibody drugs is mostly achieved by constructing expression vectors *in vitro* through genetic engineering technology. Given that recombinant antibodies often need to undergo a series of post-translational modifications (such as glycosylation modification), folding, and correct cleavage, antibody drugs with biological activity and low immunogenicity can be produced, therefore, mammalian cells have become the dominant system for the production of recombinant antibodies, especially for full-length monoclonal antibodies (Hussain et al., 2021; Kim et al., 2021; Ha et al., 2022).

**FIGURE 2:**
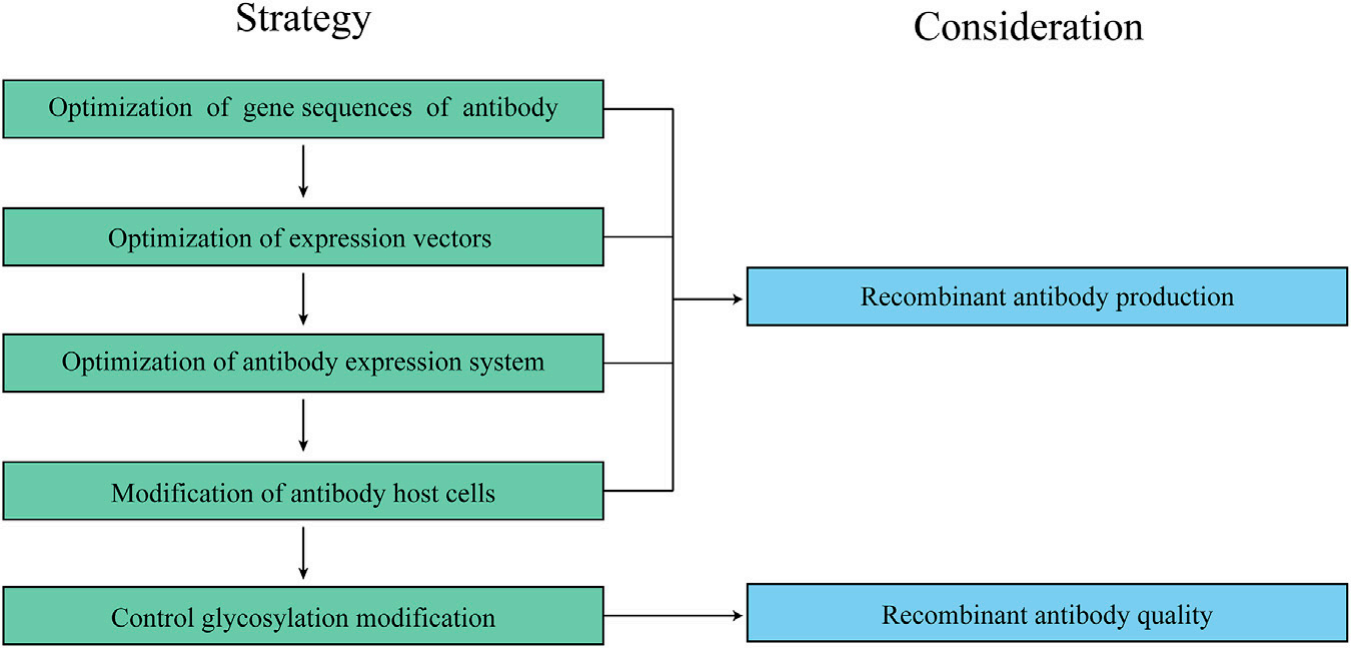
A workflow for optimizing recombinant antibody production and quality in CHO cells. Too many factors can impact recombinant antibody production and quality in CHO cells, therefore, it is important to follow a specific workflow when dealing with recombinant antibody production and quality.

Compared with *Escherichia coli* and other expression systems, the molecular structure and glycosylation type of recombinant antibodies produced by mammalian cell expression systems are similar to natural antibodies, and mammalian cells can be cultured in suspension or serum-free medium on a large scale (Kunert and Reinhart, 2016). Chinese hamster ovary (CHO) cells are the preferred system for the production of recombinant antibodies in mammalian cell expression systems (Ritacco et al., 2018; Gupta et al., 2021). In 1986, the first recombinant therapeutic protein, tissue plasminogen activator (tPA), was approved for marketing (Kaufman et al., 1985), its expression titer was less than 50 mg/L. After 30 years of rapidly development and technological breakthroughs, progress in therapeutic antibody field has been accelerated, the antibody expression titers have increased more than 100–200 times, even the yield of therapeutic antibody has exceeded 10 g/L (Li et al., 2010; Weng et al., 2020). The approved therapeutic monoclonal antibodies produced in CHO cells until 2020 are listed in Table 1. However, the low expression level of the CHO cells system and high investments during large-scale cell culture lead to high costs for producing recombinant antibody drugs (Donaldson et al., 2021; Torres and Dickson, 2021). Therefore, to improve the productivity and quality of recombinant antibodies, researchers need to optimize and design the gene sequences of antibody and expression vectors, to optimize antibody expression system and transform the host cell lines of antibodies, and to control glycosylation modification, for upgrading key parameters during industrial production and promoting the healthy development of recombinant antibody drugs and even the biomedical industry. A workflow for optimizing recombinant antibody production and quality in CHO cells is listed in Figure 2.

**TABLE 1:** List of approved therapeutic monoclonal antibodies produced in CHO cells.

Trade name	Active ingredient	Developer/Manufacturer	Year of first approval
Rituxan	Rituximab	Genentech	1997
Enbrel	Etanercep	Immunex, now Amgen	1998
Herceptin	Trastuzumab	Genetech	1998
Campath	Alemtuzumab	Genzyme	2001
Humira	Adalimumab	Abbott	2002
Zevalin	Ibritumomab tiuxetan	Biogen-Idec Pharmaceuticals	2002
Raptiva	Efalizumab	Genzyme	2003
Xolair	Omalizumab	Genetech	2003
Avastin	Bevacizumab	Genentech	2004
Vectibix	Panitumumab	Amgen	2006
Actemra	Tocilizumab	Genentech	2009
Prolia	Denosumab	Amgen	2010
Adcetris	Brentuximab vedotin	Seattle Genetics	2011
Yervoy	Ipilimumab	Bristol-Myers Squibb	2011
Perjeta	Pertuzumab	Genentech	2012
Gazyvaro	Obinutuzumab	Roche	2013
Kadcyla	Trastuzumab emtansine	Roche	2013
Blincyto	Blinatumomab	Amgen	2014
Entyvio	Vedolizumab	Takeda	2014
Keytruda	Pembrolizumab	Merck and Co.	2014
Lemtrada	Alemtuzumab	Sanofi	2014
Opdivo	Nivolumab	Bristol-Myers Squibb	2014
Sylvant	Siltuximab	Janssen	2014
Cosentyx	Secukinumab	Novartis	2015
Darzalex	Daratumumab	Janssen	2015
Nucala	Mepolizumab	GlaxoSmithKline	2015
Praluent	Alirocumab	Sanofi, Regeneron	2015
Praxbind	Idarucizumab	Boehringer-Ingelheim	2015
Repatha	Evolocumab	Amgen	2015
Lartruvo	Olaratuumab	Eli Lilly	2016
Taltz	Ixekizumab	Eli Lilly	2016
Tecentriq	Atezolizumab	Roche	2016
Zinplava	Bezlotoxumab	Merck and Co.	2016
Bavencio	Avelumab	Merck KGaA, Pfizer	2017
Dupixent	Dupilumab	Regeneron, Sanofi	2017
Fasenra	Benralizumab	AstraZeneca	2017
Hemlibra	Emicizumab	Genentech	2017
Imfinzi	Durvalumab	AstraZeneca	2017
Kevzara	Sarilumab	Sanofi, Regeneron	2017
Ocrevus	Ocrelizumab	Roche	2017
Siliq	Brodalumab	Valeant	2017
Tremfya	Guselkumab	Janssen	2017
Aimovig	Erenumab	Amgen	2018
Ajovy	Fremanezumab	Teva	2018
Cablivi	Caplacizumab	Sanofi	2018
Crysvita	Burosumab	Ultragenyx	2018
Emgality	Galcanezumab	Eli Lilly	2018
Ilumya	Tildrakizumab	Sun Pharma	2018
Libtayo	Cemiplimab	Sanofi, Regeneron	2018
Lumoxiti	Moxetumomab pasudotox	Innate Pharma	2018
Takhzyro	Lanadelumab	Shire	2018
Trogarzo	Ibalizumab	TaiMed Biologics	2018
Ultomiris	Ravulizumab	Alexion	2018
Adakveo	Crizanlizumab	Novartis	2019
Beovu	Brolucizumab	Novartis	2019
Evenity	Romosozumab	Amgen	2019
Polivy	Polatuzumab	Genentech	2019
Skyrizi	Risankizumab	AbbVie	2019
Blenrep	Belantamab	GlaxoSmithKline	2020
Danyelza	Naxitamab	Y-mAbs Therapeutics	2020
Ebanga	Ansuvimab	Ridgeback Biotherapeutics	2020
Enspryng	Satralizumab	Roche	2020
Margenza	Margetuximab	MacroGenics	2020
Monjuvi	Tafasitamab	MorphoSys, Incyte	2020
Sarclisa	Isatuximab	Sanofi	2020
Tepezza	Teprotumumab	Horizon	2020
Trodelvy	Sacituzumab	Gilead	2020
Uplizna	Inebilizumab	Viela Bio	2020
Vyepti	Eptinezumab	Lundbeck	2020

## Optimization of Gene Sequences of Antibody

### Control the Proportion of Light and Heavy Chains

**FIGURE 3:**
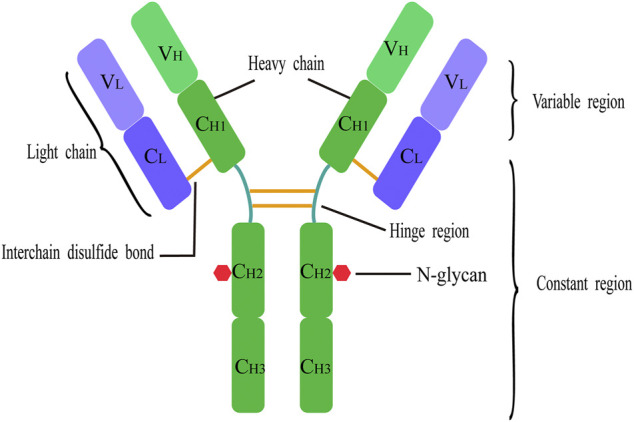
Basic structure of antibody. Each IgG antibody molecule consists of four polypeptide chains (two identical light chains and two identical heavy chains joined by disulfide bonds) and has two antigen-binding sites. Each light chain and each heavy chain consists of a variable region and a constant region. Each heavy chain consists of a variable domain (VH) and three or four constant domains (CH); each light chain consists of a variable domain (VL) and a single constant domain (CL). Human IgG structure with glycans attached at Asn297 N-glycosylation site in the CH2.

Most IgG molecules have a symmetrical structure consisting of four polypeptide chains including two heavy chains (HC) and two light chains (LC) (Figure 3). In the endoplasmic reticulum, the antibody binding protein (BiP) briefly binds to the heavy-chain polypeptide of the antibody, and the light chain is secreted out of the cell as polymerized dimers. Before assembling into a tetramer, BiP maintains a binding state with the heavy-chain polypeptide. Once the light-chain polypeptide is lacking, the heavy chain will not be secreted outside the cell, causing the proteasome in the cell to be unable to degrade the heavy-chain polypeptides secreted to extracellularly; thus, the unit productivity of antibody is significantly reduced (Vanhove et al., 2001; Ha et al., 2019). Therefore, excess light-chain polypeptides should be ensured to improve the expression of antibodies. In general, in the traditional process of antibody preparation, researchers often construct light- and heavy-chain genes of antibodies into two vectors to further express the antibody molecules. The disadvantage of this method is that the ratio of light and heavy chains is not controllable. In addition, the ratio of light and heavy chains affects the quality of monoclonal antibodies, such as the formation of aggregates and glycosylation modification. Chung et al. confirmed that excessive heavy-chain polypeptides may be the main cause of polymerization (Chung et al., 2018). In transient expression, the ratio of polypeptides can be controlled by adjusting the relative amount of each vector. However, this method is difficult to operate because the integration of plasmids is often random, and the number of gene copies and integration sites cannot be controlled artificially (Schlatter et al., 2005). Therefore, the industrial production of antibody drugs requires stable transfection of cell lines and proper control of the ratio of light and heavy chains, which are particularly important to achieve high expression, low polymerization, and consistent N-glycosylation profile of antibodies.

### Change of Gene Arrangement

The heavy- and light-chain genes of antibodies use different vectors and then enter the host cells by co-transfection. This method has very low requirements on the vector, but the transfection efficiency is high, which is a common strategy for recombinant antibody expression (Carrara et al., 2021). However, the main defect of this strategy lies in the random integration of light- and heavy-chain genes into chromosomes, and the insertion positions and numbers are different, resulting in uncontrollable expression levels of light and heavy chains (Ahmadi et al., 2016). The second arrangement is to construct an independent expression unit of light and heavy chains on the same vector; the light and heavy chain genes use separate promoters and polyadenylic acid (Poly A) tail sequences of different lengths. This strategy can theoretically achieve equal mole expression levels of light- and heavy-chain genes. However, such adjacent reading frames may cause transcriptional interference, resulting in the imbalanced transcription of light and heavy chain and ultimately inhibiting the expression of recombinant antibodies (Davies et al., 2011). Based on the advantages and disadvantages of the two gene arrangements, researchers adopted a third arrangement, in which an internal ribosome enter site (IRES) or 2A peptide in the same open reading frame to connect light-chain genes, namely, triscistronic expression vector. This strategy can effectively avoid the occurrence of transcription interference and control the proportion of light and heavy chain expression. When expressing monoclonal antibodies using IRES-mediated tricistronic vector, the IRES connects heavy-chain genes, light-chain genes, and resistance genes for expression on the same vector. This strategy can maintain over 70% productivity of positive clones and 2-fold increase in the yield of recombinant antibodies (Li et al., 2007; Yeo et al., 2018). Ho et al. designed four IRES-mediated tricistronic vectors to control the ratio of light and heavy chains, depending on the different positions of the light and heavy chains and selection marker genes on the vector; they also compared the expression level and quality of antibodies, including aggregate formation, N-glycosylation, and conformational stability. The results show that excess light chain is essential for high expression of antibody and also reduces the occurrence of polymerization. In addition, the main cause of polymerization is that the excessive heavy chains cannot effectively fold with the limited light chains and can change the N-glycosylation and reduce the conformational stability (Ho et al., 2013b). Controlling the ratio of light and heavy chains is crucial for the expression and quality of monoclonal antibodies under stable transfection conditions (Ho et al., 2012). In particular, excessive light chains cannot only achieve high antibody expression but also reduce polymerization and maintain low fragmentation levels, which are very beneficial for obtaining high-yield antibodies (Ho et al., 2013b). However, the fragment length of IRES itself is relatively large, which occupies more space, and the expression of IRES upstream and downstream antibody molecules is seriously imbalanced (Bayat et al., 2018). In addition, the activity of IRES is not easy to adjust, which will affect the biological activities of other expressed proteins. In recent years, a prominent approach is based on self-cleavage 2A peptide-mediated multi-gene construction method (Chng et al., 2015; Van der Weken et al., 2019). This strategy effectively avoids the disadvantage of low expression of downstream genes caused by the simultaneous expression of multiple genes and is commonly used in multi-gene expression (Luke and Ryan, 2018; Li et al., 2020). Ho et al. compared the effects of F2A and IRES on the production of a monoclonal antibody in CHO-DG44 cells. The expression level of the monoclonal antibody produced by F2A-mediated tricistronic vector was significantly higher than that of IRES-mediated vector under transient or stable transfection conditions; the expression level of the recombinant antibody was affected by the position of light- and heavy-chain cistrons (Ho et al., 2013a).

### Codon Optimization

Antibody molecules usually have the molecular structure of tetramer glycoprotein. Light- and heavy-chain genes in antibody molecules need to have coordinated expression to biosynthesize tetramer IgG. However, when expressing recombinant antibodies, the special secondary structure in the gene sequence and other factors often lead to low expression of antibody, which can be remedied by codon optimization strategy. Codon optimization involves gene synthesis, gene transcription, mRNA translation, etc., with the ultimate goal of efficiently expressing recombinant antibodies (Ayyar et al., 2017; Mauro and Chappell, 2018; You et al., 2018). Before expressing a gene, the rare codon in the gene should be searched first. If there are too many rare codons, then the translation rate of antibody will be affected. The expression of the target gene in transgenic hosts can be increased by selecting the codon preferred by the receptor without changing the amino acid sequence (Mauro, 2018). When the codon of the variable region of an antibody is replaced with the preferred codon of the natural human antibody gene, the antibody expression levels in mammalian cells are significantly increased by two- to 3-fold (Carton et al., 2007).

### Signal Peptide Optimization

Signal peptide is a key factor for the secretion of recombinant antibodies, and the high-efficiency expression of these antibodies is closely related to the signal peptide (Haryadi et al., 2015; Wang et al., 2016). When expressing recombinant antibodies, in addition to the use of their own signal peptides, the following strategies are often applied: ① replace the signal peptide sequence of the efficiently expressed secreted proteins (Attallah et al., 2017); ② modifying the primary structure of the signal peptide sequence in the original antibody; ③ replace the protein signal peptide sequence in some viruses; however, viral vectors are generally not recommended when considering the safety of the expressed recombinant antibodies; and ④ select the preferred host codon. When selecting a suitable signal peptide, the preferred codons of the expression host should be fully considered, and the signal peptide should be further optimized. Ramezani et al. showed a 2-fold increase of the pertuzumab production in CHO cells by optimizing codons and selecting appropriate signal peptide strategies (Ramezani et al., 2017).

## Construction and Optimization of Expression Vectors

The construction of high-efficiency expression vectors is considered an important strategy to improve the expression level of recombinant antibodies. The expression level of the target genes in mammalian cells is mainly affected by the state of chromosome region integrated by the gene of interest, the copy number of the target gene, and its transcription and translation efficiency (Hung et al., 2010; Gupta et al., 2019; Carver et al., 2020; Hoseinpoor et al., 2020). Therefore, vector construction strategy should consider optimizing the integration site and position effect on the chromosome and improving the transcription and translation efficiency to effectively improve the expression level of recombinant antibodies.

### Optimization of Integration Site and Position Effect

The integration site status of target gene on the mammalian cell chromosome plays a decisive role in its expression level and its stability in host cells. Only clones formed by cells whose integration sites are in transcription active region of chromosomes can express the target gene at a high level. However, transgene silencing often occurs due to the random integration of the target gene after transfer into cells (Hilliard and Lee, 2021). During the expansion of cell culture, the promoter methylation will lead to the attenuation of expression. Therefore, selecting an appropriate promoter and optimizing the combination of promoters and different regulatory elements can improve the expression of recombinant antibodies and increase the stability of expression. Some chromatin-modifying elements can prevent transgene silencing, including matrix attachment regions (MAR) (Buceta et al., 2011; Mohammadian et al., 2019; Zhang et al., 2020), locus control regions (LCR) (Sharma et al., 2019; Morgan et al., 2020), ubiquitous chromatin opening elements (UCOEs) (Pfaff et al., 2013; Harraghy et al., 2015; Nematpour et al., 2017; Rocha-Pizaña et al., 2017), stabilizing anti-repressor elements (STAR) (Kwaks et al., 2003; Otte et al., 2007; Van Blokland et al., 2007), and insulators (Benabdellah et al., 2014; Chetverina et al., 2014; Naderi et al., 2018; Pérez-González and Caro, 2019). Moreover, artificial chromosome expression (ACE) and targeted integration technology, including Flp-In and recombinase-mediated cassette exchange system (RMCE), can overcome the shortcomings of random integration (Kennard et al., 2009; Soler et al., 2018; Reinhart et al., 2019; Ng et al., 2021). The introduction of these functional elements into the vector construction can greatly increase the proportion of high-expression clones and shorten the construction cycle of engineered host cells (Table 2).

**TABLE 2:** Optimization strategies of integration site and position effect for improving recombinant antibody expression.

Element	Mechanism of action
MAR	A boundary element, which acts as an insulator and overcomes the position effect
LCR	A DNA sequence composed of many regulatory elements such as enhancers or isolators, which has the function of stabilizing the loose structure of chromatin and controlling the sequential expression of individual genes at the locus
UCOE	A class of non-methylated CpG islands, combined with the promoter to prevent the formation of heterochromatin and gene silencing
STAR	Regulatory factors that can block the repressor protein, increase transgene expression and expression stability
Insulator	A class of enhancer blocking elements prevent the heterochromatin marker from spreading to the euchromatin region
ACE	Pre-engineered artificial chromosomes, which allows for the targeted transfection of single or multiple genes and eliminates the need for random integration into native host chromosomes
Flp-In	Site-directed integration of target genes into transcriptionally active regions
RMCE	Recombinase-mediated site-directed integration technology

### Improvement of Transcription and Translation Efficiency

Transcription is the initial step of gene expression, among which promoters, enhancers, and transcription termination signals have very important impacts on transcription efficiency and mRNA stability. CMV promoters are considered to be one of the strongest viral promoters, and hEF-1α promoters have the stronger transcription initiation efficiency and are more suitable for large-scale production of recombinant antibodies (Ebadat et al., 2017). In recent years, the construction of synthetic or heterozygous promoters will become an effective strategy to improve the transcription efficiency (Patel et al., 2021).

Enhancers can improve the transcription efficiency and can function over long distances. Constructing heterozygous enhancers is a good strategy to improve transcription efficiency and obtain high transcriptional activity. Xu et al. used the CMV promoter and CA hybrid promoter to insert the SV40 enhancers downstream of SV40 Poly A; compared with the CMV promoter, cells driven by the CA hybrid promoter can increase the production of target proteins by two times (Xu et al., 2001).

Introns can increase the expression of foreign genes. Xu et al. compared the effects of five different introns on transgene expression in CHO cells. Under transient and stable transfection conditions, the SV40 intron can obtain the highest transgene expression level among five introns, which can also obtain high-level of recombinant protein production in CHO cells (Xu et al., 2018).

The regulation of translation level and processing efficiency of translation products will also significantly affect the expression of target gene. Eisenhut et al. developed the 5′-untranslated region (UTR) RNA-structures to impact translation efficiency, further systematically tune protein expression levels in mammalian cells and eventually help to optimize recombinant protein expression (Eisenhut et al., 2020). Vivirius et al. found a universal translation enhancer element in the 5′-end non-translation area of Hsp70 mRNA, which can enhance the translation efficiency of the cap-dependent structure (Vivinus et al., 2001).

## Optimzation of Antibody Expression System

### Gene Amplification Screening System

At present, the two common gene amplification screening systems are dihydrofolate reductase (DHFR) and glutamine synthetase (GS) (Budge et al., 2021; Huhn et al., 2021). The CHO-DHFR amplification system mainly uses the CHO cell line of DHFR defect to obtain high yields of recombinant antibodies in the presence of methotrexate (MTX). Akbarzade-sharbaf et al. used DHFR system to express a therapeutic antibody (Trastuzumab); after seven rounds of MTX, the total antibody valence can be as high as 50–60 mg/L/day (Akbarzadeh-Sharbaf et al., 2013). As another mature and widely used system, the GS amplification system is an explicit gene amplification selection marker, which usually requires pressure and stable selection of two rounds of methionine sulphoximine (MSX) to obtain high-expression cell lines and reduce the screening time. However, the two systems have some defects, such as the decline in the antibody production after long-term screening; therefore, these systems need to be further optimized. The strategy of using low activity of GS as the screening marker could obtain high-yield clones after MSX removal, further efficiently increase the antibody production (Lin et al., 2019).

### Screening Marker Weakening

Two strategies can be employed to weaken the screening marker. One is to mutate the screening marker and reduce its activity. Neomycin-phosphotransferase (NPT) is one of the commonly used screening markers in eukaryotic expression systems. The second strategy is to reduce the expression of the screening marker. Noguchi et al. started the transcription of DHFR through the late promoter of weak promoter SV40 and used the method of IR/MAR-*dhfr* fusion to easily separate the cell line of high-expression recombinant protein, which further improved the production of the recombinant proteins (Noguchi et al., 2012).

## Modification of Antibody Host Cells

Many mammalian cells can produce recombinant antibodies, including CHO, NS0, and SP2/0 cells, which can produce non-human glycosylated modification. Owing to the difference between the growth and metabolic characteristics of animal cells, the expression level and modification ability of recombinant antibody are also different (Dhara et al., 2018). Therefore, we can further transform the host cells, improve the expression level by engineering cell line, and meet the production capacity and quality requirement of antibody drugs as much as possible (Lu et al., 2018; Liu et al., 2021; Zhou et al., 2021).

### Glycosylation Engineering

The enzymes responsible for glycosylation can be modified in non-humanized cells, so the expressed recombinant antibody is more similar to the natural humanized protein in terms of glycosylation level and type, thereby improving the biological effect of recombinant antibody. Glycosylation mainly includes genetic and non-genetic modification strategies. The glycosylation of protein can affect the pharmacological activity and pharmacodynamics of the antibody, so researchers can optimize the recombinant antibodies through genetic engineering to achieve the glycosylation modification of cell lines (Yang Y et al., 2021). For example, the therapeutic activity of recombinant IgG3 antibodies was significantly improved after transfecting murine 2, 6-sialyltransferase into CHO cells (Jassal et al., 2001).

### Anti-Apoptosis

Anti-apoptosis is a hot spot in the current host cell transformation strategy, and the overexpression of anti-apoptotic genes is one of the common strategies. Effective anti-apoptotic genes include Bcl-2, Bcl-xL, and so on. Lee et al. achieved the overexpression of Bcl-2 and Beclin-1 in CHO-DG44 cells; the results showed that the time of cell culture became longer, the cell survival rate was significantly improved, and the occurrence of apoptosis was suppressed (Lee et al., 2013). Kim et al. overexpressed Bcl-xL in recombinant CHO cells, which can improve cell survival rate, prolong culture time, and inhibit apoptosis and autophagy by inhibiting the activation of caspase-3and caspase-7 (Kim et al., 2009).

### Cellular Metabolic Engineering

In mammalian cell culture, the metabolic engineering strategy changes the cell metabolism pathway, which can effectively promote cell growth and product synthesis, discover more novel metabolite additives, and reduce the accumulation of metabolic inhibitors (Toussaint et al., 2016; Gupta et al., 2017; Fouladiha et al., 2020; Yao et al., 2021). In cells with low lactate/glucose, the expression levels of lactate dehydrogenase and the accumulation of lactate are reduced, resulting in an increase in the synthetic products. Zhou et al. used siRNA technology to reduce the expression levels of lactate dehydrogenase A (LDHa) and pyruvate dehydrogenase kinase (PDHK) genes, reducing the lactate levels by 90% and increasing the production of therapeutic monoclonal antibodies (Zhou et al., 2011). In addition, the production of recombinant antibodies can be multiplied by several times by effectively blocking engineered cells in the G_1_ phase.

### Cellular Cycle Regulation Engineering

In the large-scale cell culture process, with the continuous exploration of the regulatory mechanism of cell cycle, researchers have applied cell cycle regulation genes to cell proliferation control. Fussenegger et al. found that p21, p27, and p53 are cell cycle G_l_/S suppressor proteins; after CHO cells express these proteins, they can prevent cells from entering the S phase, keep the growth in a static state, and increase the production of secreted alkaline phosphatase (SEAP) to 10–15 times (Fussenegger et al., 1998).

## Control Glycosylation Modification

Recombinant antibodies are biological macromolecular drugs, and the normal performance of their biological functions is inseparable from the complex post-translational modification process. As the most important type of post-translational modification of recombinant antibodies, glycosylation modification has certain effects on the biological activity of antibody, immunogenicity, *in vivo* metabolism, antibody-dependent cytotoxicity, and complement-dependent cytotoxicity. Therefore, glycosylation modification of antibody molecules has been widely used in the development of novel antibody drugs.

### Type of Antibody Glycosylation Modification

According to different connection modes, antibody glycosylation is usually divided into N-linked glycosylation and O-linked glycosylation. The Fc fragments of two heavy chains in the monoclonal antibody molecule contain an N-glycosylation modification site at the 297th aspartic acid. The N-glycosylation modification of monoclonal antibody drugs usually presents biantennary sugar chains, and sometimes fucosylation and sialylation may occur (Szabo et al., 2022). In addition, some monoclonal antibody drugs undergo O-glycosylation modification. For example, a humanized monoclonal antibody drug produced by CHO cells has O-glycosylation modified by a single glucose molecule (Tanaka et al., 2013). At present, most of the antibody drugs on the market are produced by CHO cells. CHO cells can produce antibodies that are close to the glycotype of human serum antibodies; however, the glycosylation of antibodies produced by most engineered cell lines is different from that of human serum antibodies. For example, antibodies produced from mouse-derived animal cells have a high proportion of fucose modifications and a low proportion of galactosylation modifications (An, 2009). In contrast to human cells, CHO cells lack the expression of *α*-2,6-sialyltransferase, while only express *α*-2,3-sialyltransferase (Jenkins et al., 1996; Lin et al., 2015; Chung et al., 2020). Consequently, CHO cells inherently cannot produce glycoproteins with similar terminal sialic acid content as compared to human cells (Yin et al., 2017). Furthermore, CHO cells lack N-acetylglucosamine transferase (GnT-III) expressed by human cells, resulting in the differences in the modification of N-acetylglucosamine glycosylation from human cells (Butler, 2005). These findings indicate that different cell types produce different types of antibody glycosylation (Attallah et al., 2020; Dyukova et al., 2021; Zhao et al., 2021). In summary, the type of glycosylation modification of monoclonal antibody drugs is closely related to the production system, selected cell line, and incubation process.

### Control Strategy of Antibody Glycosylation Modification

**FIGURE 4:**
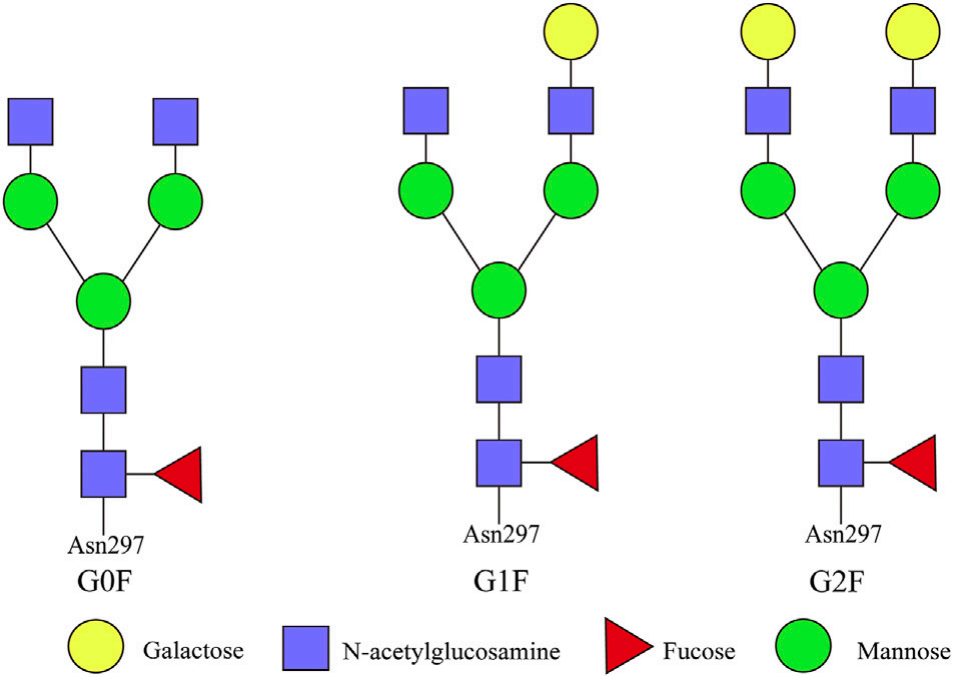
Types of glycosylation modification of recombinant antibodies. The representative N-glycan structures identified on antibody Fc fragment are G0F, G1F, and G2F. G0F: asialo, agalactose, biantennary complex, core substituted with fucose; G1F: asialo, mono-galactosylated, biantennary complex, core substituted with fucose; G2F: asialo, galactosylated, biantennary complex, core substituted with fucose.

Although the mass of sugar chains only accounts for 2% of the total molecular weight of antibody, the sugar chains on the Fc and Fab fragments play a very important role in the affinity, structural maintenance, metabolism, and immunogenicity of the antibody molecule (Krapp et al., 2003; Chung et al., 2008; Alessandri et al., 2012). The physiological activity of therapeutic antibodies is mainly mediated by two mechanisms: one is mediated by the affinity between the variable region of the antibody and antigen, causing the neutralization or apoptosis of the target antigen, which is mainly by means of the Fab fragment of antibody to recognize and bind antigenic substances; the other is the immune effect mediated by the Fc fragment of antibody, including antibody-dependent cell-mediated cytotoxicity (ADCC) and complement-dependent cytotoxicity (CDC) (Salinas-Jazmín et al., 2022). Metabolic analysis and mathematical model can be used to analyze the production process to control the type and degree of glycosylation modification of recombinant antibodies to reduce the immunogenicity of antibody drugs and optimize their effector function (Sha et al., 2016). The types of glycosylation modification of recombinant antibodies are shown in Figure 4.

Sialylation modification: Sialylatedglycans are the key components of glycoproteins. According to relevant studies, monoclonal antibody sialylation can inhibit inflammatory response and reduce cell toxicity by means of different receptors in the Fc fragment (Scallon et al., 2007). The anti-inflammatory activity of the over-sialylated monoclonal antibody obtained by affinity chromatography purification will be significantly enhanced. Moreover, the higher degree of monoclonal antibody sialylation is closely related to a decrease in ADCC activity (Zhang et al., 2019; Nguyen et al., 2020).

Core fucosylation modification: Related studies have shown that core fucose is an important glycosylation structure that affects the ADCC activity of recombinant antibodies (Matsumiya et al., 2007). Most recombinant IgG produced by CHO cells have core fucose in their Fc sugar chains (Zimmermann et al., 2021). To improve the binding activity of the low-affinity receptor IIIa (FcγR3a) of the antibody to the Fc fragment as well as ADCC activity, researchers have adopted a variety of strategies to reduce the level of fucosylation of IgG, including the use of *α*-1,6-fucose cell lines in which *α*-1,6-fucosetransferase gene is knocked out (Davies et al., 2001; Wang et al., 2018).

Galactosylation modification: Approximately 95% of recombinant IgG produced by CHO cells contains galactose as the terminal sugar. Terminal galactosylation plays an important role in the conformation of the Fc fragment, and the core fucosylation modification alone has slight effect on the conformation of the Fc fragment (Kotidis et al., 2019). The existing results indicate that galactosylation at the end of the Fc fragment will seriously affect the CDC activity of IgG, and the reduction of its glycosylation level will also weaken the CDC activity (Wei et al., 2021).

Mannosylation modification: The content of a high-mannose structure should be minimized as much as possible during the production of recombinant antibodies due to the high immunogenicity of high-mannose structures. In addition, the content of mannose in the Fc fragment of the antibody varies greatly between different cultured cells and different batches (Brantley et al., 2021). Recombinant antibodies have two clearance pathways in organisms; one is the asialoglycoprotein receptor in the liver that binds and mediates the clearance, and the other is to bind to mannose receptors on the surface of macrophages in the liver. The high-mannose structure antibody molecules in the Fc fragment can be quickly eliminated from the plasma, further reducing the efficacy of recombinant antibodies (Liu, 2015).

Expression of GnT-III: Studies have shown that the bisected glycosylated epidermal growth factor receptor (EGFR) monoclonal antibody is prepared by introducing the GnT-III gene and highly expresses bisecting acetylglucosamine residues; this strategy can increase the ADCC activity by 3 times and increase the anti-proliferative activity by 1.36 times, and almost no *α*-Gal was detected. The bisecting EGFR monoclonal antibody prepared by glycosylation engineering contains only a small amount of *α*-Gal, which greatly improves the biological activity *in vitro*. At present, this study has not been further verified *in vivo* (Jenkins and Curling, 1994; Yi et al., 2014).

## Conclusion and Future Perspectives

In recent years, with the development and application of proteomics technology, the development of large-scale culture of animal cells for antibody drug production has devolved from the simple optimization of some process parameters to the recent omics research, e.g., transcriptomics, proteomics, metabolomics, glycomics, and fluxomics. The complex metabolic network of production cells and production mechanism of recombinant antibodies have gradually become clear. Raab et al. developed a cell line through genetic engineering by a novel bottom-up microRNA (miRNA) screening approach for optimizing the production and secretion of therapeutic antibodies (Raab et al., 2022). At present, the expression level and quality control of recombinant antibodies has always been one of the important bottlenecks restricting the development of antibody drugs.

The high-efficiency expression and quality of recombinant antibodies can be affected by multiple factors, which can be achieved by genetic engineering, including the optimization of antibody gene sequence, construction of efficient expression vector, optimization of antibody expression system, modification of antibody host cells, and glycosylation site modification (Table 3). The application of these optimization strategies can effectively shorten the time of antibody generation and improve the expression of target antibodies. However, different optimization strategies have advantages and disadvantages, and how to effectively integrate these optimization strategies to make it an efficient operation system needs further study. Under different process conditions, the differences in the yield of recombinant antibodies by the production cell lines can be analyzed from the levels of genomics, proteomics, and metabolomics, laying a solid foundation for large-scale cell culture processes. In the future, the large-scale production of recombinant antibodies and development of culture processes will develop rapidly in the direction of stabilizing production capacity and improving quality. Furthermore, big data and multi-omics technologies are also beneficial to provide new research directions, more and more process analytical technologies are applied to cell culture processes, which will provide more ideas for improving the efficiency of recombinant antibodies. Therefore, the focus shifts towards how to control the quality of recombinant antibody drugs scientifically and rationally, researchers should further combine the clinical evaluation and post-market safety monitoring, and continue to explore quality control.

**TABLE 3:** Basic strategies for improving recombinant antibody production and quality in CHO cells.

Action stage	Strategy
Processing assembly	Light and heavy chain expression balance, host cell modification
Integration site	Screening marker weakening, chromosome location screening
Gene optimization	Change of gene arrangement, codon optimization
Gene dose	The amplifiable screening marker gene weakening
Transcription	The potent promoter, enhancer and intron, appropriate antibody gene structure
Translation	Translational enhancer and the regulation of translation products
Post-translational modification	Control of antibody glycosylation modification
Secretion	The suitable antibody secretion signal peptide
Other	Choose the suitable host cells, modify host cells and try to remove non-productive clones
